# Supplementary material for: SPARC promotes insulin secretion through down-regulation of RGS4 protein in pancreatic β cells
Source: Sci Rep. 2020 Oct 16;10:17581. doi: 10.1038/s41598-020-74593-w (PMC7567887; doi:10.1038/s41598-020-74593-w)
Supplement: Supplementary file 1 — Supplementary file1 [file 41598_2020_74593_MOESM1_ESM.pdf]

SPARC promotes insulin secretion through down-regulation of RGS4 protein in pancreatic β cells

Li Hu[1, 2, 3], Fengli He[1, 2, 3], Meifeng Huang[1, 2,3], Qian Zhao[4], Lamei Cheng[4], Neveen Said[5], Zhiguang Zhou[1, 2, 3], Feng Liu[1, 2, 3, 6], and Yan-Shan Dai[1, 2, 3, 7, 8]

[1] Department of Metabolism and Endocrinology, The Second Xiangya Hospital, Central South University, Changsha, Hunan, China.

[2] National Clinical Research Center for Metabolic Disease, The Second Xiangya Hospital, Central South University, Changsha, Hunan, China.

[3] Metabolic Syndrome Research Center, The Second Xiangya Hospital, Central South University, Changsha, Hunan, China.

[4] Institute of Reproductive and Stem Cell Engineering, Central South University, Changsha, Hunan 410011, China

[5] Department of Cancer Biology, Wake Forest University School of Medicine, Winston-Salem, NC, USA

[6] Department of Pharmacology, University of Texas Health Science Center at San Antonio, San Antonio, Texas, USA

[7] Current Address: Bristol-Myers Squibb Company, Princeton, NJ, USA.

[8] Corresponding author:

Ydai88@csu.edu.cn

Supplemental Figures

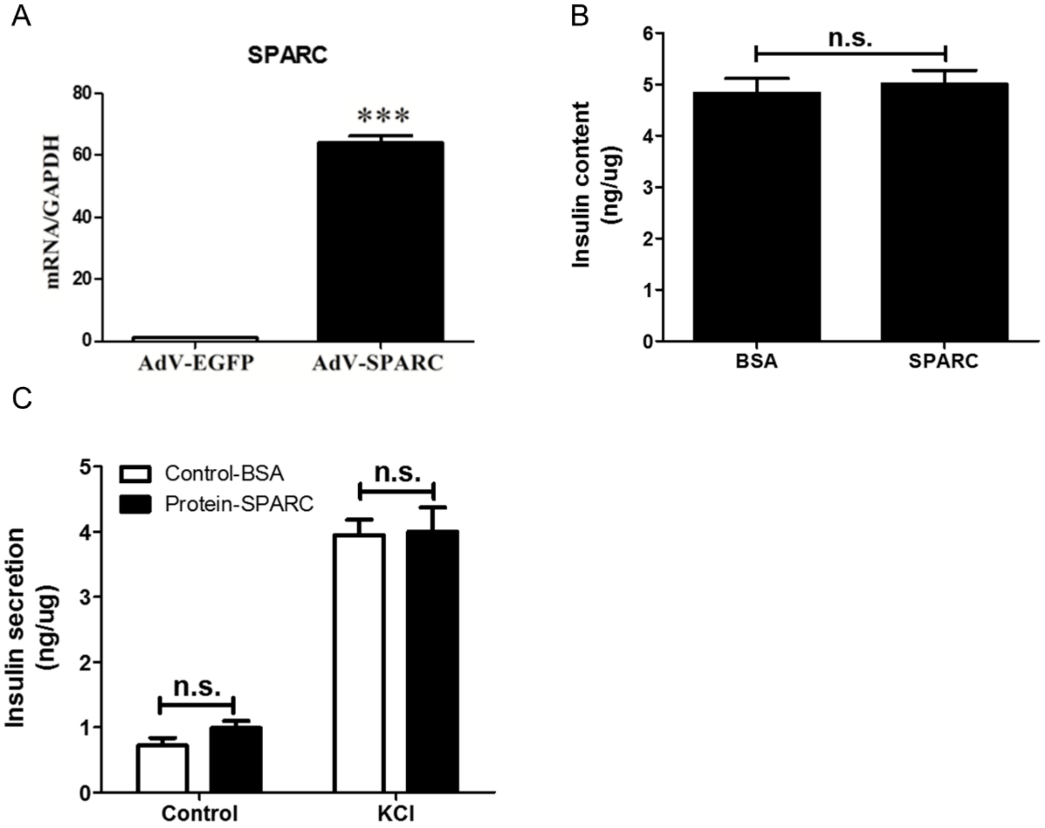

Supplemental Fig. 1.

(A) Isolated mouse islets were infected with AdV-EGFP or AdV-SPARC for 24 hours. Relative SPARC mRNA levels were quantified by RT-qPCR and normalized with GAPDH. *** denotes $P < 0.001$, n=3. Statistical significance was assessed by unpaired two-tailed Student's t test. (B) Mouse islets were cultured in RMPI1640 medium with 11 mM glucose. Groups of islets were then incubated with Krebs Ringer Bicarbonate buffer and with 16.7 mM glucose and 100 µM Oxo-M in the presence or absence of SPARC for 1 hour. The islet pellets in each group were lysed with acid-ethanol (0.18 M HCl in 75% ethanol) overnight at -20°C, followed by centrifugation and supernatant collection. The insulin level in the supernatants was determined with insulin ELISA kit. Shown were the levels of insulin secretion normalized to protein concentration. Statistical significance was assessed by unpaired two-tailed Student's t test. n.s. denotes $P > 0.05$, n=3. (C) Mouse islets were cultured in RMPI1640 medium with 11 mM glucose. Groups of islets were then incubated with Krebs Ringer Bicarbonate buffer and with 16.7 mM glucose in the presence or absence of recombinant SPARC, 1 µg/ml, or 16.7 mM glucose and 40 mM KCl in the presence or absence of SPARC for 1 hour. The supernatants were collected, and insulin was determined with insulin ELISA kit. Shown were the levels of insulin secretion normalized to protein concentration. n.s. denotes $P > 0.05$, n=3. Statistical significance was assessed by unpaired two-tailed Student's t test.

A

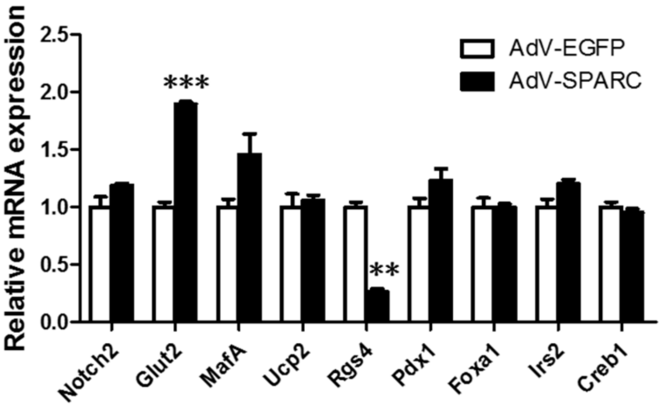

B

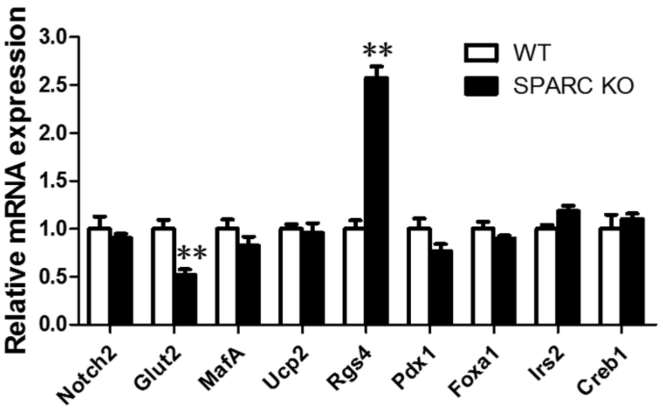

Supplemental Fig. 2.

(A) Min6 cells were infected with AdV-EGFP or AdV-SPARC for 24 hours. Total RNAs were prepared from AdV-EGFP and AdV-SPACR treated Min6 cells and were subjected to RT-qPCR and normalized with GAPDH. ** denotes $P < 0.01$, n=3. *** denotes $P < 0.001$, n=3. Statistical significance was assessed by unpaired two-tailed Student's t test. (B) Mouse islets were isolated from WT or sparc -/- mice and relative gene expression levels were quantified by RT-qPCR and normalized with GAPDH. ** denotes $P < 0.01$, n=3. Statistical significance was assessed by unpaired two-tailed Student's t test.

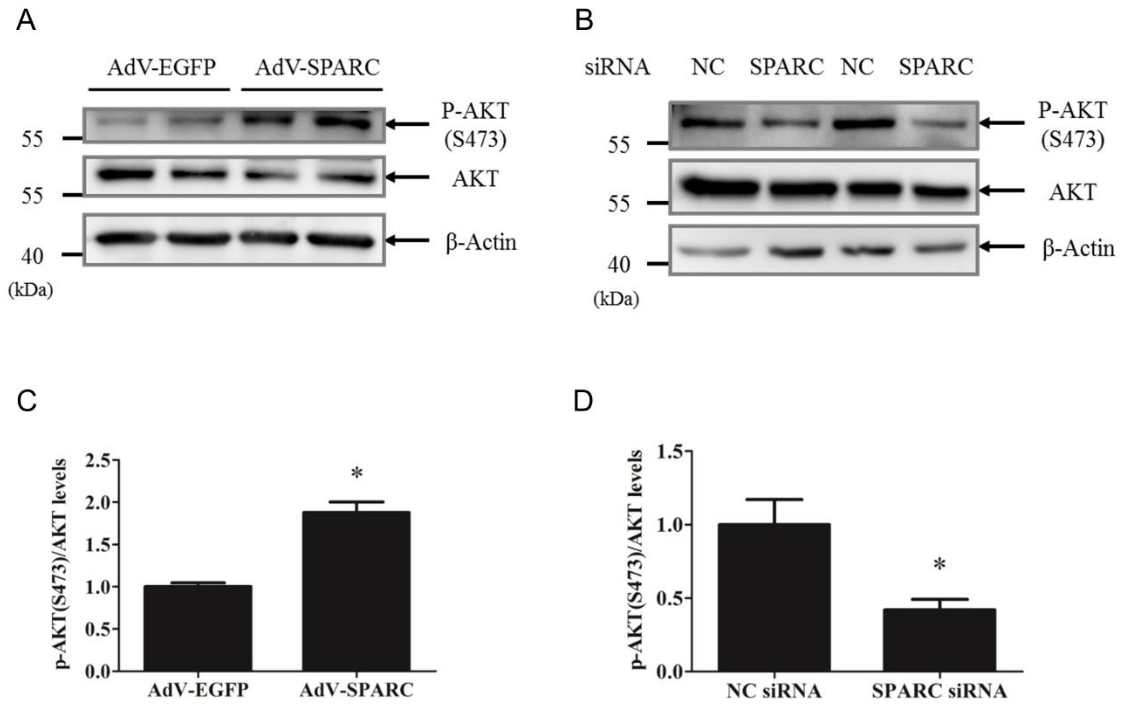

Supplemental Fig. 3.

(A, C) Overexpression of AdV-SPARC in Min6 cells increased AKT S473 phosphorylation. Min6 cells were infected with AdV-EGFP or AdV-SPARC for 24 hours. The cell lysates were subject to Western blotting with anti-p-AKT (473) or anti-β-actin antibody. Lower panel was ratio of p-AKT S473 /total AKT quantified from three experiments (C). (B, D) Knocking down of SPARC in Min6 cells decreased AKT S473 phosphorylation. Min6 cells were transfected with SPARC siRNA and cultured for 48 hrs. The cell lysates were subjected to Western blotting with anti-p-AKT(S473) antibody or anti-beta-actin antibody (B). Lower panel was ratio of p-AKT S473 /total AKT quantified from three experiments (D). Statistical significance was assessed by unpaired two-tailed Student’s t test. * denotes $P < 0.05$, n=3.

A

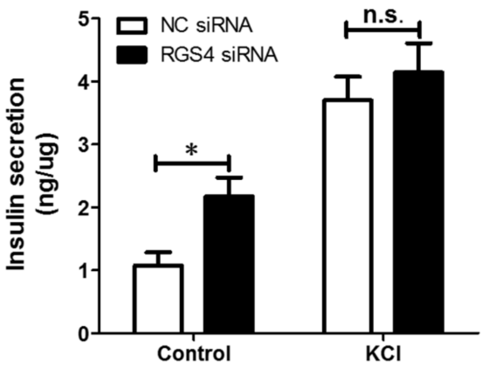

Supplemental Fig. 4.

(A) Min6 cells were transfected with RGS4 siRNA and cultured for 48 hours. Groups of Min6 cells were then incubated with Krebs Ringer Bicarbonate buffer with 16.7 mM glucose, or 16.7 mM glucose and 40 mM KCl for 1 hour. The supernatants were collected, and insulin was determined with insulin ELISA kit. Shown were the levels of insulin secretion normalized to protein concentration. n.s. denotes $P > 0.05$, n=3. * denotes $P < 0.05$, n=3. Statistical significance was assessed by unpaired two-tailed Student's t test.

Original imaging of the Western Blot gels

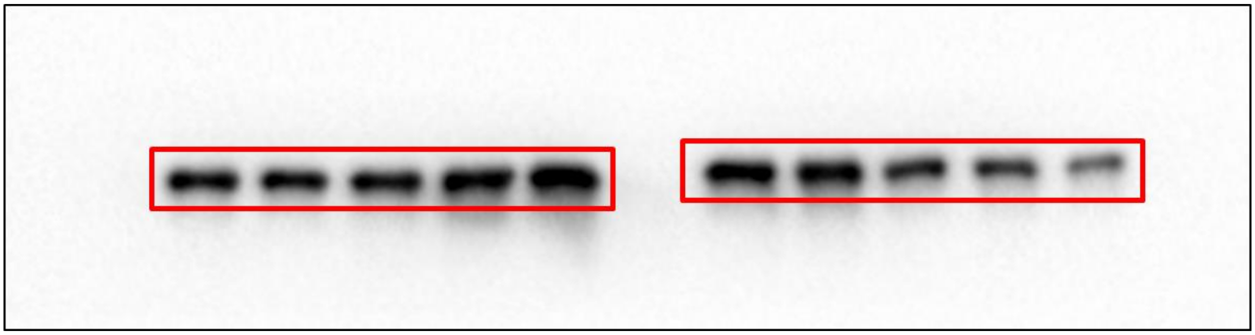

Full unedited gel for Figure 1A and 1C top, SPARC expression

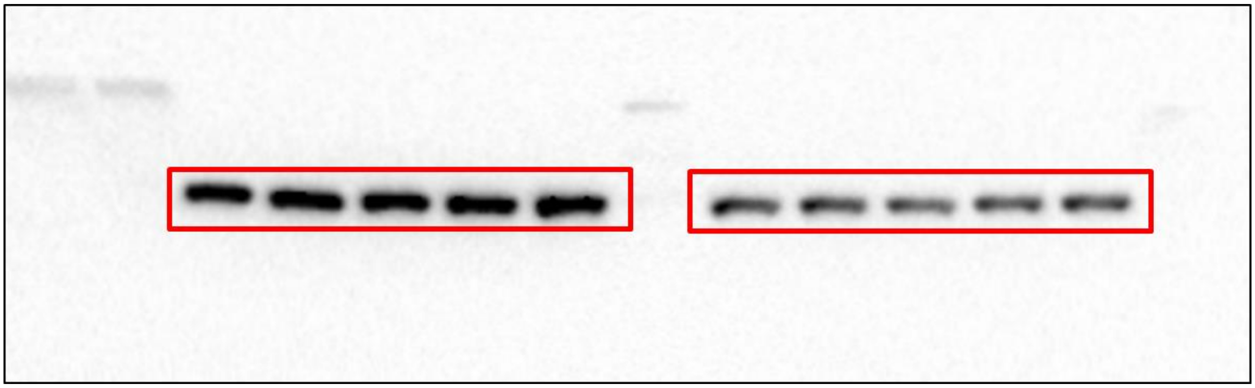

Full unedited gel for Figure 1A and 1C bottom, β-Actin expression

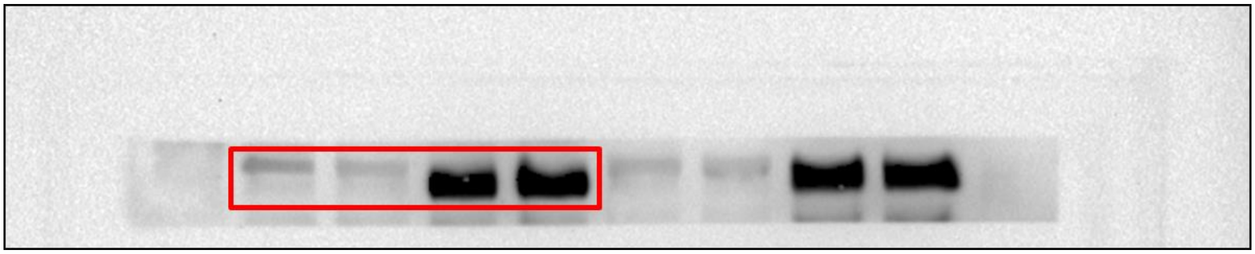

Full unedited gel for Figure 3C top, SPARC expression

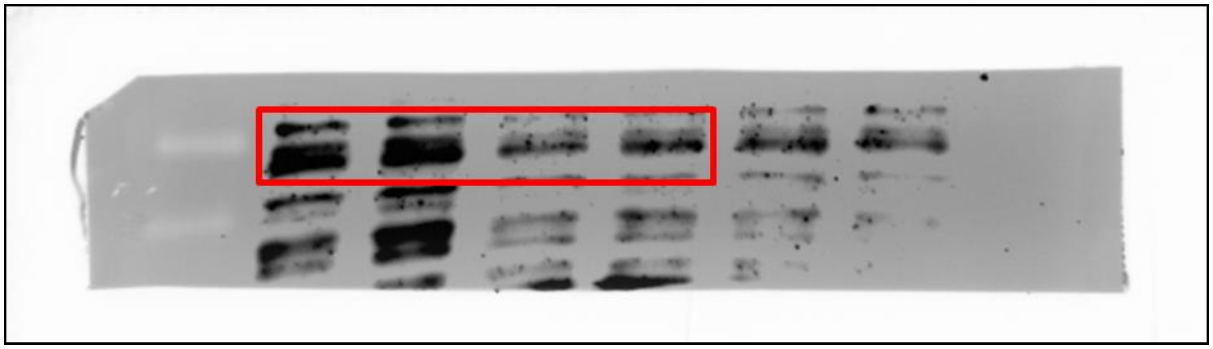

Full unedited gel for Figure 3C middle, RGS4 expression

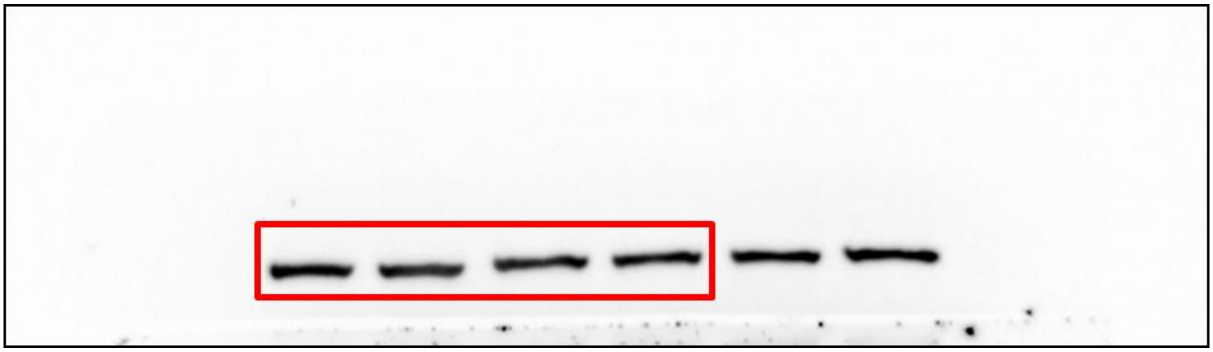

Full unedited gel for Figure 3C bottom, β-Actin expression

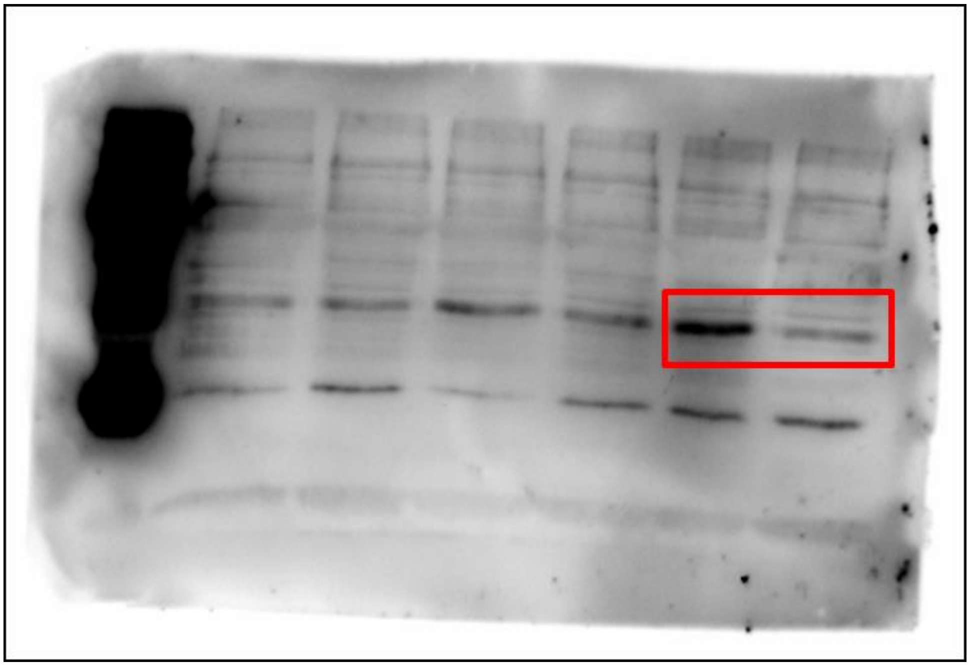

Full unedited gel for Figure 4A top, SPARC expression

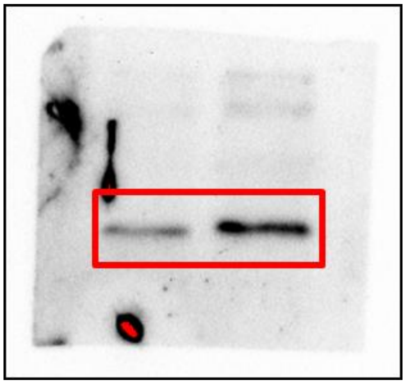

Full unedited gel for Figure 4A middle, RGS4 expression

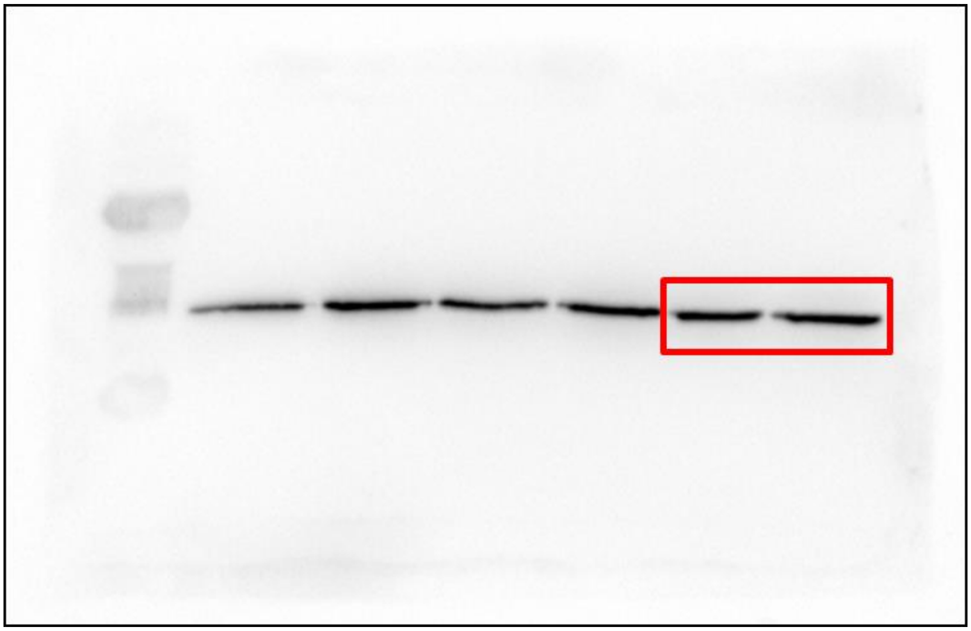

Full unedited gel for Figure 4A bottom, β-Actin expression

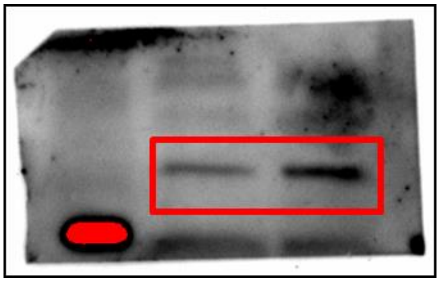

Full unedited gel for Figure 4C top, RGS4 expression

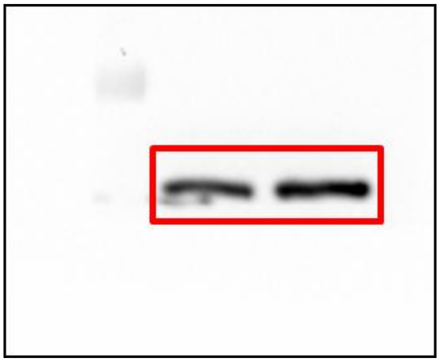

Full unedited gel for Figure 4C bottom, β-Actin expression

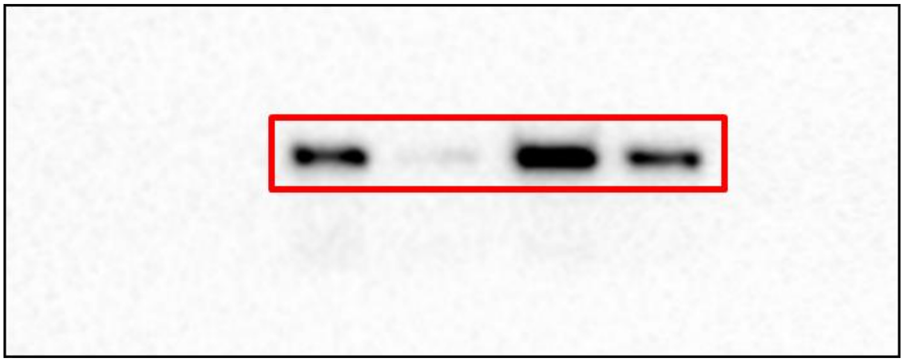

Full unedited gel for Figure 5A top, P-AKT (S473) expression

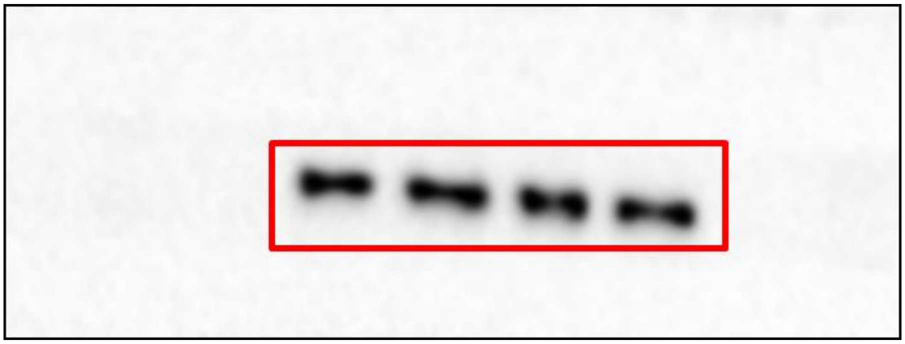

Full unedited gel for Figure 5A middle, AKT expression

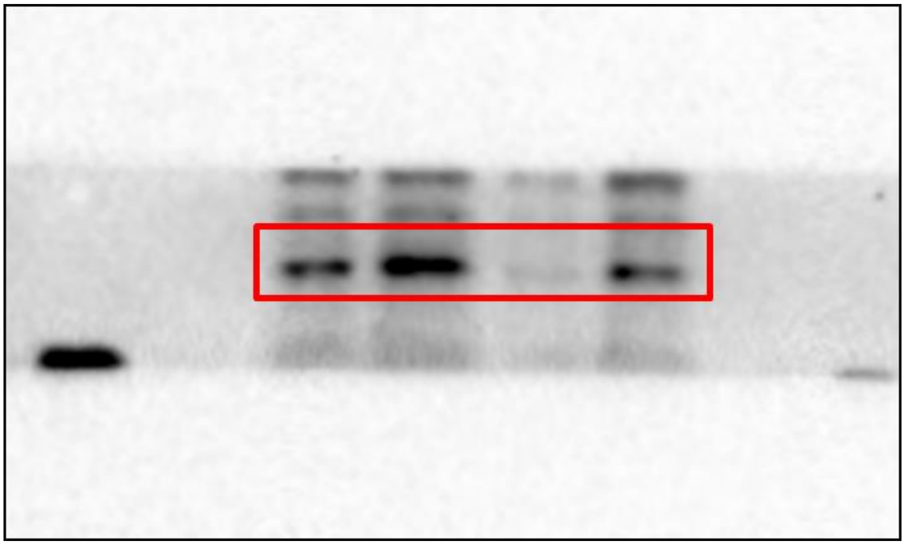

Full unedited gel for Figure 5A middle, RGS4 expression

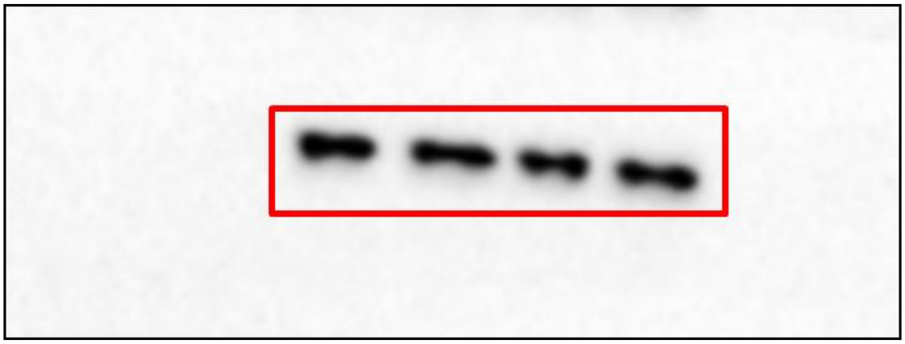

Full unedited gel for Figure 5A bottom, β-Actin expression

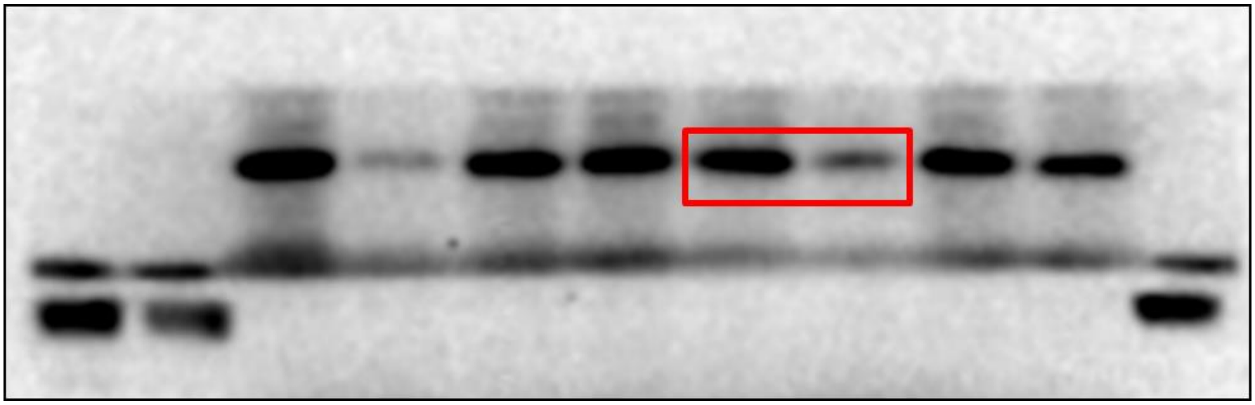

Full unedited gel for Figure 6C top, RGS4 expression

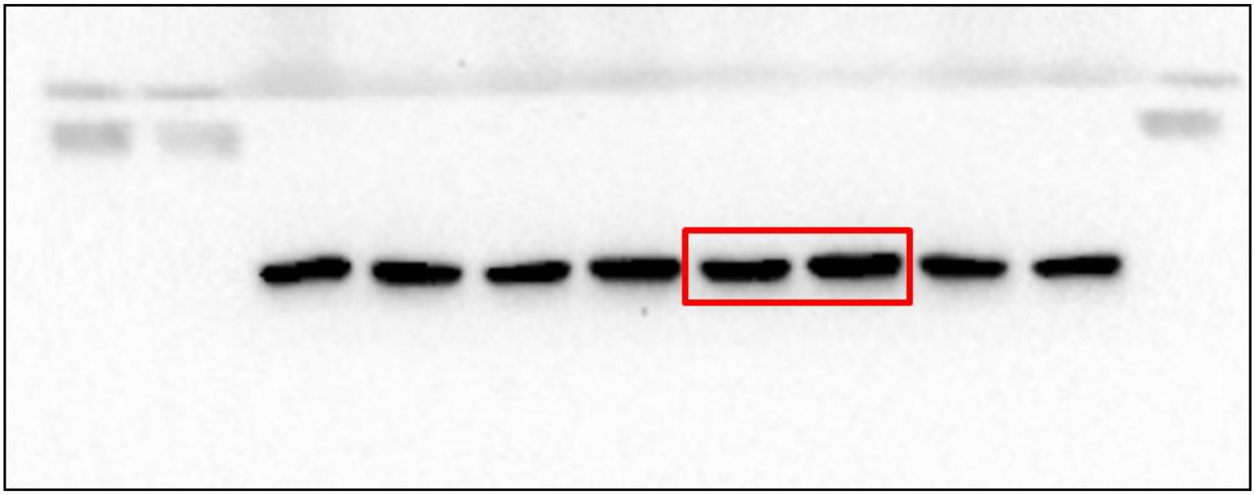

Full unedited gel for Figure 6C bottom, β-Actin expression

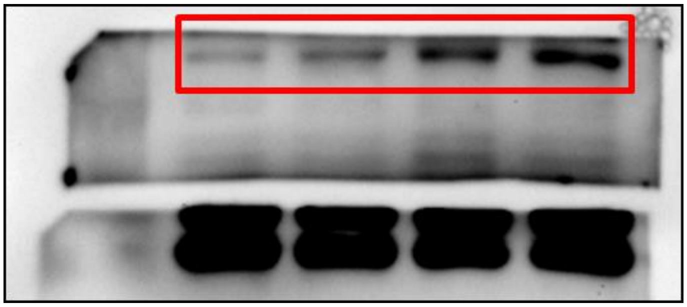

Full unedited gel for Supplemental Figure 3A top, P-AKT (S473) expression

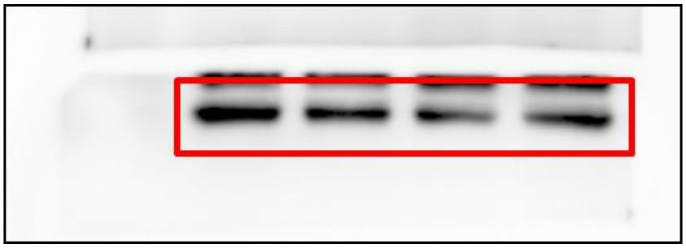

Full unedited gel for Supplemental Figure 3A middle, AKT expression

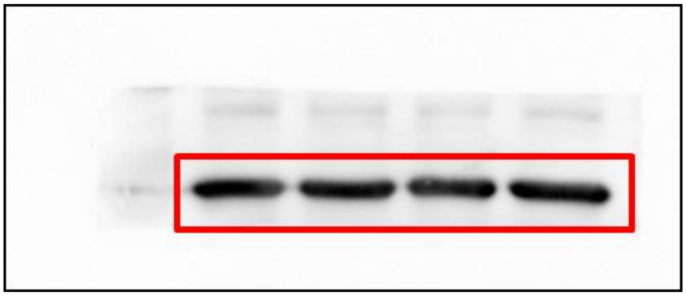

Full unedited gel for Supplemental Figure 3A bottom, β-Actin expression

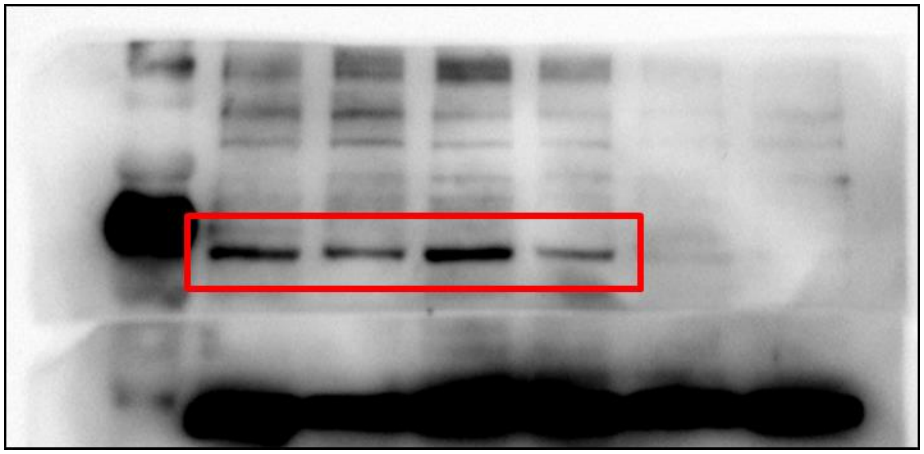

Full unedited gel for Supplemental Figure 3B top, P-AKT (S473) expression

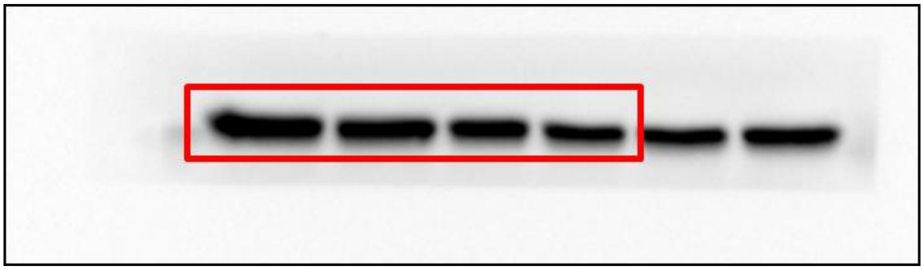

Full unedited gel for Supplemental Figure 3B middle, AKT expression

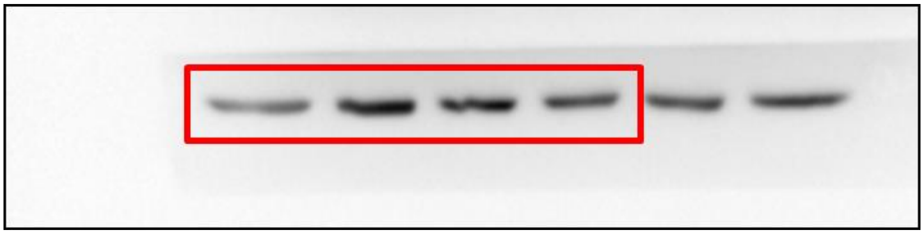

Full unedited gel for Supplemental Figure 3B bottom, β-Actin expression
